# Systematic Review and Meta-Analysis: Malnutrition and In-Hospital Death in Adults Hospitalized with COVID-19

**DOI:** 10.3390/nu15051298

**Published:** 2023-03-06

**Authors:** Mona Boaz, Vered Kaufman-Shriqui

**Affiliations:** Department of Nutrition Sciences, Ariel University, Kiryat Hamada 3, Ariel 40700, Israel

**Keywords:** malnutrition, COVID-19, in-hospital mortality, meta-analysis

## Abstract

Background: Malnutrition and increased malnutrition risk are frequently identified in hospitalized adults. The increase in hospitalization rates during the COVID-19 pandemic was accompanied by the documentation of adverse hospitalization outcomes in the presence of certain co-morbidities, including obesity and type 2 diabetes. It was not clear whether the presence of malnutrition increased in-hospital death in patients hospitalized with COVID-19. Objectives: To estimate the effect of malnutrition on in-hospital mortality in adults hospitalized with COVID-19; and secondarily, to estimate the prevalence of malnutrition in adults hospitalized with malnutrition during the COVID-19 pandemic. Methods: EMBASE, MEDLINE, PubMed, Google Scholar, and Cochrane Collaboration databases were queried using the search terms malnutrition and COVID-19 and hospitalized adults and mortality. Studies were reviewed using the 14-question Quality Assessment Tool for Studies with Diverse Designs (QATSDD) (questions appropriate for quantitative studies). Author names; date of publication; country; sample size; malnutrition prevalence; malnutrition screening/diagnostic method; number of deaths in malnourished patients; and number of deaths in adequately nourished patients were extracted. Data were analyzed using MedCalc software v20.210 (Ostend, Belgium). The Q and *I*^2^ tests were calculated; a forest plot was generated, and the pooled odds ratio (OR) with 95% confidence intervals (95%CI) were calculated using the random effects model. Results: Of the 90 studies identified, 12 were finally included in the meta-analysis. In the random effects model, malnutrition or increased malnutrition risk increased odds of in-hospital death by more than three-fold: OR 3.43 (95% CI 2.549–4.60), *p* < 0.001. The pooled prevalence estimate for malnutrition or increased malnutrition risk was 52.61% (95% CI 29.50–75.14%). Discussion and Conclusions: It is clear that malnutrition is an ominous prognostic sign in patients hospitalized with COVID. This meta-analysis, which included studies from nine countries on four continents with data from 354,332 patients, is generalizable.

## 1. Introduction

Malnutrition, or more specifically, undernutrition, can be defined as the adverse clinical, functional, metabolic, and anthropometric outcomes of nutrient deficiency [1]. Malnutrition can also be the result of disease and can interact with the disease to produce unfavorable outcomes [2]. While prevalence estimates vary, malnutrition has been reliably shown to increase with age, disease, and disability [3]. 

Malnutrition prevalence estimates in older adults range from less than 5% among healthy community-dwelling seniors [4] to more than 20% among nursing home residents [5] and more than 50% among hospitalized adults [6]. Among older hospitalized adults, inadequate protein intake during hospitalization predicts in-hospital death even more strongly than inadequate energy intake [7]. Malnutrition in hospitalized adults is associated with greater in-hospital mortality compared to rates among well-nourished and obese patients [8].

Malnutrition has been associated with multimorbidity, frailty, sarcopenia, inflammation, and insulin resistance, leading to increased mortality [9]. The importance of nutrition status cannot be overstated, as evidenced by the directive from the Norwegian Directorate of Health to screen all adults for malnutrition [10]. This position paper recommends that all adults aged 18 years or older undergo evaluation for nutrition status in most healthcare environments, including in-patient and out-patient settings, as well as individuals receiving assistance with food and/or feeding. The screening tool selected by the advisory committee was the Malnutrition Screening Tool (MST), based on a systematic review of its reliability and validity.

The Global Leadership Initiative on Malnutrition (GLIM) acknowledged that while undernutrition is associated with inadequate nutrient intake and/or absorption, it is also associated with impaired health conditions such as illness, trauma and/or inflammatory states, which may lead to increased healthcare costs, functional decline, morbidity and death. Despite the universality of concern about malnutrition, a globally accepted definition of this condition, screening or diagnostic approach was not available. Thus, participants in the GLIM project endeavored to create a standardized, globally acceptable consensus on malnutrition diagnosis. GLIM has determined that malnutrition diagnosis is a two-step process which begins with screening for malnutrition using an accepted, valid malnutrition screening tool, followed by a nutrition assessment through which diagnosis and determination of severity are made [11]. 

Screening tools thus represent the first step in diagnosing malnutrition. Frequently used among these are the Mini Nutrition Assessment (MNA); the Nutrition Risk Screening 2002 (NRS2002); the Malnutrition Universal Screening Tool (MUST); Controlling Nutritional status (CONUT); and Prognostic Nutrition Index (PNI). The MNA, often used in its short form (MNA-SF), screens for malnutrition in the elderly and includes queries on food intake, involuntary weight loss, mobility, psychological stress or acute disease, neurocognitive disorders, and current BMI [12]. The NRS2002 was designed for use in hospitalized adults and includes measures of involuntary weight loss, reduced food intake, and disease severity [13]. Designed for use in the community, MUST has nevertheless been employed by many hospitals [14]. MUST considers current BMI, involuntary weight loss, and disease severity and provides nutrition management guidelines based on score [15]. CONUT utilizes n total peripheral lymphocytes, serum albumin, and total cholesterol to categorize hospitalized patients as low, medium, or high risk for malnutrition. These data can be uploaded daily, providing real-time monitoring or patient nutrition status throughout the hospital confinement [16]. Similarly, the PNI uses the equation 10 × serum albumin (g/dL) + 0.005 × lymphocyte count (per mm^3^) to develop a patient score where a lower score indicates more severe nutrition risk [17].

This plethora of diagnostic tools available gives great freedom to the clinician, but lack of between-method agreement has been documented [18]. On the other hand, validity and inter-rater reliability has been demonstrated between various methods and combinations of methods [19]. In the absence of a gold standard for screening for and/or diagnosing malnutrition, the current study will assume that the method used in a given study identified malnutrition with a reasonable degree of accuracy; however, this heterogeneity inserts variability into data summarization.

### COVID-19

Worldwide, many governments managed the COVID-19 outbreak by mandating lockdowns. These curfews, quarantines, and school and business closures had a negative psychological impact on the populations subjected to them [20]. Specifically, population surveys revealed elevated levels of psychological stress, depression, and anxiety [21], which were reported across cultures [22]. Suicidal ideation also increased during lockdowns [23], exemplified by increased calls to suicide hotlines during the same periods [24]. Indeed, an increase in suicide attempts was observed during the COVID-19 epidemic [25]. 

Associations between emotional distress, including anxiety and depression and eating behaviors are well documented. A direct association between binge eating and anxiety has been reported [26], and poor diet quality has been associated with increased depression and suicidal ideation [27]. During the COVID-19 pandemic in general and during lockdowns in particular, when anxiety, depression, and psychological stress were increased, alterations in dietary intake were noted, though the direction of that change is not entirely clear. For example, an increase in fast food and sugary food was concomitantly reported with an increase in fresh foods among UK adults with diabetes [28]. On the other hand, Korean adults, particularly seniors, experienced a worsening in diet quality during the pandemic [29], and findings from an international survey identified an association between self-reported anxiety and diet quality in community-dwelling adults [30]. 

Given the worsening in diet quality during the pandemic, it is possible that adults were hospitalized for COVID-19 with greater malnutrition risk than they might have during other historical periods. The adverse impact of malnutrition on hospitalization outcomes is well established [31]. Thus, the present meta-analysis sought to quantify the impact of malnutrition on in-hospital mortality in adult patients hospitalized with COVID-19.

The primary objective of the present study is to estimate the effect of malnutrition on in-hospital mortality in adults hospitalized with COVID; and secondarily, to estimate the prevalence of malnutrition in adults hospitalized with malnutrition. 

## 2. Methods

### 2.1. Protocol and Registration

The present meta-analysis followed the PRISMA guidelines [32]. The protocol was registered on the National Institute for Health Research (NIHR) PROSPERO site registration number CRD42023392009 [33].

### 2.2. Inclusion Criteria

Studies were eligible for inclusion in the present meta-analysis if they were observational, including cross-sectional, retrospective, or prospective designs. Study populations in the included studies were limited to adults aged 18 years or older hospitalized with COVID-19. All included articles were published in the period 2021–2023 to provide coverage of legacy and more recent COVID variants. All publications were published in the English language in peer-reviewed journals. Published and in-press articles were acceptable.

For all included studies, malnutrition was the stated exposure and in-hospital morality was the stated outcome, though not necessarily the primary outcome of the study.

In-hospital mortality was identified as an outcome (not necessarily a primary outcome).

### 2.3. Exclusion Criteria

Studies were only eligible for inclusion in the present analysis if they provided the full information necessary for data extraction needed for the analysis. Additionally, studies in which malnutrition prevalence was not stated were not included. Finally, narrative reviews, systematic reviews, and meta-analyses were not eligible for inclusion in the present meta-analysis.

### 2.4. Databases

The following databases were queried: EMBASE, MEDLINE, PubMed, Google Scholar, and Cochrane Collaboration.

### 2.5. Search Words

Search words used in each of the databases were: malnutrition and COVID-19 and hospitalized adults and mortality. 

### 2.6. Study Quality

Prior to inclusion, studies were reviewed using the 14-question Quality Assessment Tool for Studies with Diverse Designs (QATSDD) (questions appropriate for quantitative studies). Using this tool, a score on a given item can range from 0 (not at all or not mentioned) to 3 (complete). Scores for each of the items were compared between the two reviewers (MB, VKS), and agreement was defined as a score from one reviewer within one point of the other reviewer’s score. A 75% agreement was set as the minimum level of between-reviewer concordance. Disagreement beyond this was to be settled by discussion, but all articles reviewed met this criterion. Total scores were also compared between reviewers. According to protocol, an article with a total score less than 21 points (half of the total possible points overall for a quantitative study) were to be omitted. None of the articles reviewed at the full manuscript level were omitted for this reason.

### 2.7. Search Strategy

The study examined all published and in-publication articles from 2020 to 2023, in order to capture hospitalizations for COVID. Only English-language publications were included. Search terms in each of the databases included malnutrition, COVID-19, mortality, and hospitalized adults. 

### 2.8. Data Extraction

Data extracted from each included article were author names; date of publication; country; sample size; malnutrition prevalence; malnutrition screening/diagnostic method; number of deaths in malnourished patients; number of deaths in adequately nourished patients. Data were extracted to an Excel spreadsheet.

### 2.9. Data Analysis

Data were analyzed using MedCalc software v20.210 (Ostend, Belgium). The Q and *I*^2^ tests were calculated, indicating a great deal of heterogeneity between studies. Egger’s test and Begg’s test were used to assess publication bias. A forest plot was generated and the pooled odds ratio (OR) with 95% confidence intervals (95%CI) were calculated using the random effects model. 

## 3. Results

### 3.1. Selection of Studies

**Figure 1:**
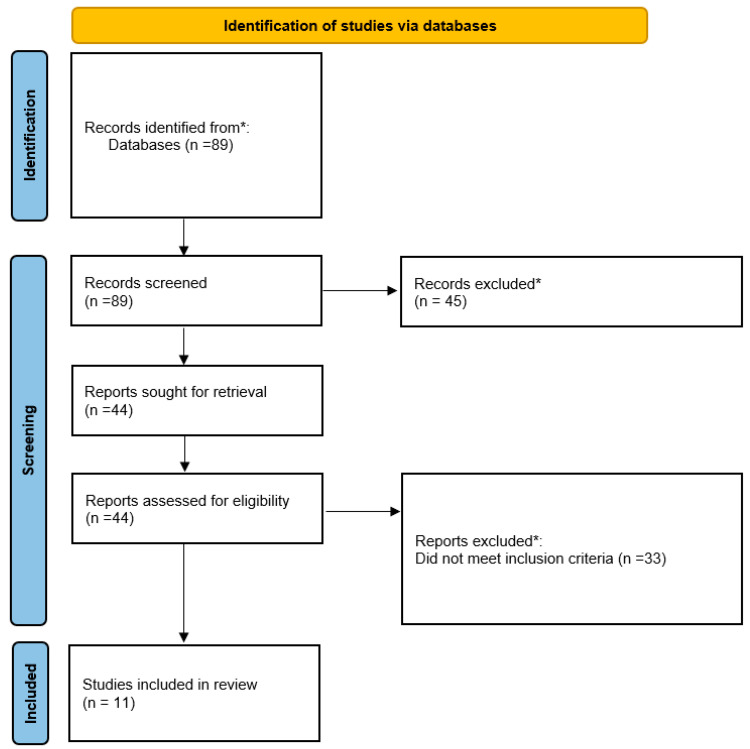
Flow diagram of study selection. * More details can be found in the article. From: Page MJ, McKenzie JE, Bossuyt PM, Boutron I, Hoffmann TC, Mulrow CD, et al. The PRISMA 2020 statement: an updated guideline for reporting systematic reviews. BMJ 2021;372:n71 [32].

The flow of study selection is presented in Figure 1.

As can be seen, 90 studies were originally identified, and their abstracts reviewed. From these, 45 studies were eliminated due to title or abstract screening. A total of 26 studies were omitted because the exposure was not malnutrition, or the outcome did not include in-hospital mortality. Another 16 studies were omitted because the study population was not appropriate: specifically, the study was conducted in children (*n* = 5); the study was conducted in non-COVID patients (*n* = 6); and the study was conducted in non-hospitalized patients (*n* = 5). Another three manuscripts were omitted because the article was not a study, but rather a set of treatment guidelines or a case report.

Of the remaining 45 studies that were retrieved for full text review, 22 were eliminated because the exposure was not malnutrition and/or the outcome was not in-hospital death. Another three were conducted in non-hospitalized individuals; seven were judged to be off topic, for example, a study designed to understand barriers to physician evaluation of nutrition status; and one was a meta-analysis. 

### 3.2. Description of Included Studies

The included studies are summarized in Table 1. A total of twelve studies were included, of which two each were from the USA, Turkey, and Brazil; and one each were from China, Switzerland, Iran, Italy, Spain, and Sweden.

Of the studies, five were prospective (of which one was historical prospective); three were retrospective; and four were cross sectional.

The included studies were published between April 2021 and December 2022. 

Sample sizes ranged from 75 to 343,188. Of the 354,332 total participants, 34,437 (unweighted fixed percentage, 9.7%) were classed as malnourished or at increased risk of malnutrition.

All studies were conducted in hospitalized adults diagnosed with COVID-19; however, COVID-19 was not necessarily the reason for hospitalization. All of the studies reported malnutrition prevalence. 

Methods of diagnosing malnutrition risk or malnutrition itself included: NRS 2002 (*n* = 6 studies); modified NRS 2002 (*n* = 1 study); MNA-SF (*n* = 2 studies); MUST (*n* = 2 studies); SGA (*n* = 1 study); PNI (*n* = 1 study); anthropometric or biochemical measures (*n* = 2 studies); CONUT (*n* = 1); diagnostic code (*n* = 1); self-reported food reduction (*n* = 2). Of these, five studies incorporated more than one method of screening for/diagnosing malnutrition.

**Figure 2:**
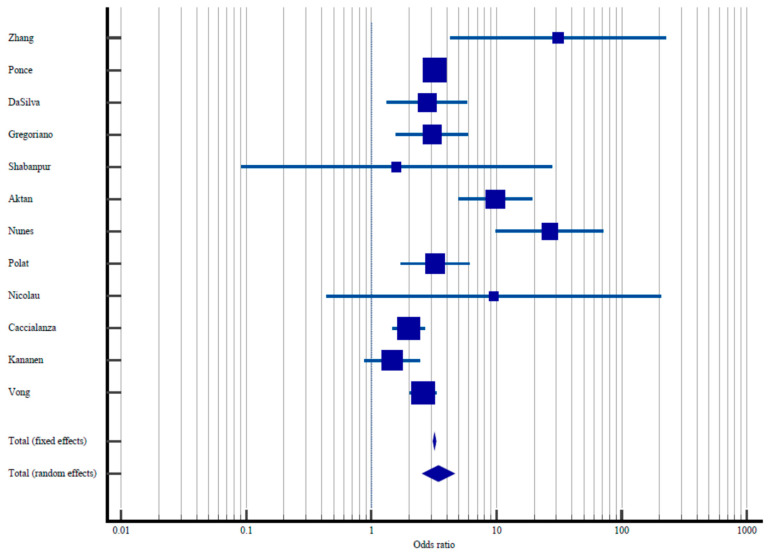
The influence of malnutrition on in-hospital death: odds ratios.

The meta-analysis of the impact of malnutrition on risk of in-hospital death is summarized in Table 2. The Q and *I*^2^ statistics indicate a high degree of heterogeneity between studies: Q = 56.5% and *I*^2^ = 80.53%, 95% CI 66.91–888.54%, *p* < 0.001. For this reason, the random effects model is used (though the fixed effects model is also presented). The odds for in-hospital mortality in patients with malnutrition range from the non-significant OR 1.46 in the Kananen et al., study to OR 26.58 in the Nunes et al., study and OR 31.010 in the Zhang et al., study. The Kananen study was retrospective and conducted in geriatric hospitals in Sweden. The Zhang et al., study was prospective, while the Nunes et al., study was cross-sectional. Three studies did not have significant OR values in the random effects model: Shabanpur et al., (OR 1.58, 95% CI 0.09–27.55); Nicolau et al., (OR 9.51, 95% CI 0.44–205.69; and Kananen et al., (OR 1.46, 95% CI 0.0.88–2.42). Both the Shabanpur et al., and the Nicolau et al., studies were cross-sectional, while the Kananen et al., study, as stated previously, was retrospective. Overall, in the random effects model, OR 3.43 (95% CI 2.55–5.40), *p* < 0.001, indicating that increased malnutrition risk or a diagnosis of malnutrition nearly quadruples the risk of in-hospital death. The forest plot of the association between malnutrition and in-hospital death is presented in Figure 2.

Publication bias was not present according to Egger’s test: intercept = 0.21, 95% CI −1.47–1.89, *p* = 0.79; and Begg’s test: Kendall’s Tau = 0.24, *p* = 0.27.

Malnutrition prevalence is summarized in Table 3. Malnutrition prevalence estimates range from 7.843 % (95% CI 7.753–7.934) in the Ponce et al., study to 96.750 (95% CI 94.507–98.258) in the Shabanpur et al., study. The *I*^2^ statistic indicates a high degree of between-study heterogeneity, necessitating the use of the random effects model: *I*^2^ = 99.93%, 95% CI 99.92–99.94%, *p* < 0.001. The pooled prevalence estimate using the random effects model is 52.61% (95% CI 29.50–75.14%). 

Egger’s test indicated publication bias: intercept = 31.82, 95% CI 15.09–48.55, *p* = 0.002. Though Begg’s test was not significant: Kendall’s Tau −0.125, *p* = 0.58, the findings nevertheless suggest publication bias. 

## 4. Discussion

The purpose of the present study was to estimate the impact of malnutrition on in-hospital mortality in adults hospitalized with COVID-19. The pooled random effects model indicates that malnutrition almost quadruples the odds of in-hospital mortality in this population, supporting the study hypothesis.

The analysis indicates that malnutrition increases odds of death by more than three-fold. This contrasts with the odds of death reported in the meta-analysis by Abate et al, who found that malnutrition increased odds of death by a factor of 10 (OR 10.14, 95% CI 6.49–15.82) [46], significantly higher than the odds of death found in the present report (OR 3.43 (95% CI 2.55–5.40). 

The influence of malnutrition on mortality in patients with COVID-19 was among the associations examined in a large, U.S. retrospective cohort study designed to identify risk factors for adverse outcomes of COVID-19, employing the National Inpatient Sample (NIS) 2020. Part of the Healthcare Cost and Utilization Project, the NIS 2020 includes data on patients hospitalized from all healthcare payers and includes unweighted data from approximately 7 million hospitalizations annually on a national level, providing data on more than 97% of the U.S. population. This study identified that malnutrition approximately doubled risk of mortality in patients with COVID-19, a risk increase similar to that of stroke and liver disease, and almost twice that of the increased risk conferred by obesity, uncomplicated diabetes and chronic obstructive pulmonary disease [47]. This finding is of particular interest given the amount of attention devoted to the risk of overweight and obesity in patients with COVID-19. In a prospective study of almost 200 consecutive adults hospitalized with COVID-19, body composition was measured using bioipedence analysis together with anthropometric and biochemical measures of inflammation and immune response. While measures of abdominal fatness increased risk for ventilatory support, it did not predict mortality [48]. An umbrella review of systemic reviews and meta analyses on the association of obesity and mortality in patients with COVID-19 detected an increased risk of 1.14–3.52 in 16 of 24 included studies, but no increase in risk in eight studies. The authors concluded that the high degree of bias prevented making definitive statements about this association [49].

Malnutrition diagnosed using bioimpedance vector analysis (BIVA) was shown to more than quadruple the risk for adverse outcomes including mechanical ventilation and 60-day mortality in adults with COVID-19 pneumonia [50].

Elevated malnutrition risk and diagnosed malnutrition have been shown to increase risk of in-hospital mortality and early post-discharge mortality in a number of disease states. For example, a retrospective study of elderly patients (mean age > 80 years) hospitalized in an acute care geriatric department defined malnutrition as MNA score < 17. At the three-month follow-up point, malnutrition was associated with increased risk of death: OR 3.519, 95% CI: 1.254–9.872, *p* = 0.017. The MNA score also predicted incident geriatric syndrome, discharge location (home vs. institution) and functional status [51].

Malnutrition also predicted short term post-hospital discharge mortality. In a northern Italian cohort of 1451 consecutively enrolled adult patients (median age 80 years) admitted to internal medicine departments in a large tertiary care hospital, nearly 16% of participants died within four months of hospital discharge. Malnutrition was defined as BMI < 18.5 kg/m^2^. Using this definition, investigators found that malnutrition more than doubled the risk of post-discharge mortality compared to patients with normal or elevated BMI. Among patients who died in-hospital, 3.2% were classed as having malnutrition compared to 8% of patients who were discharged home. This surprising finding was explained by the investigators as a reflection of the efficacy of the nutrition intervention provided to patients with malnutrition, improving survival [52]. It is also possible that the use of BMI < 18.5 kg/m^2^ as the measure of malnutrition explains these findings. If individuals with BMI in the normal, overweight and obese ranges have elevated malnutrition risk or a diagnosis of malnutrition, they will be misclassified as adequately nourished or at minimal risk. Further, if overweight and obesity are associated with improved survival, as has been demonstrated in acutely and chronically ill patient populations [53,54,55], then the estimated in-hospital mortality rate will be underestimated.

Malnutrition does not predict mortality in all patient populations. A study of the association between malnutrition risk screened using the NRS 2002 and in-hospital mortality was conducted in patients admitted to intensive care units following a cardiac arrest. This retrospective study found no difference in either the NRS 2002 score or BMI between patients who survived and those who expired; however, both serum albumin and total cholesterol levels, both markers of malnutrition, were significantly lower in patients who died. Nevertheless, the NRS 2002 score did not predict in-hospital mortality in patients after cardiac arrest [56].

Included studies were characterized by a great deal of heterogeneity, evidenced by the very large *I*^2^ values. The heterogeneity in malnutrition prevalence undoubtedly reflects the variability in malnutrition risk screening and malnutrition diagnosis methods. Further, this heterogeneity in prevalence likely contributes to the between-study heterogeneity in odds of in-hospital death. Other potential contributors to malnutrition prevalence estimate variability may be patient characteristics associated with malnutrition risk, such as advanced age and number of comorbidities.

Differences in odds estimates for death can arise from the diversity of malnutrition ascertainment methods employed in the included studies. Different screening and/or diagnostic tools can lead to differences in malnutrition prevalence estimates as well as differences in estimates of the impact of malnutrition on mortality [57]. However, if misclassification for exposure is non-differential (the probability of classifying a well-nourished individual as a malnourished individual is approximately equal to the probability of classifying a malnourished individual as a well-nourished individual), the result is bias toward the null hypothesis. This would imply that if there is an effect on the risk estimates, it is that the pooled OR is an underestimate of true risk.

The prevalence-estimate of 57.145% is somewhat greater than the prevalence estimate reported in the meta-analysis by Abate et al., which included studies of malnutrition published between 2019 and 2020 and estimated prevalence to be 49.11% (95% CI: 31.67 to 66.54) [46]. While this difference is not significant, it may nevertheless reflect changes in hospitalized patient mix as the pandemic continued. Indeed, it has been reported that the hospitalized patient mix skewed toward more seriously debilitated individuals as the pandemic progressed [58]. Additionally, it is possible that the prevalence of malnutrition in study participants is biased, since hospital personnel may have avoided the more prolonged contact with these highly contagious patients necessary to perform a thorough nutrition status assessment. Indeed, health care worker stress in caring for patients hospitalized with COVID-19 has been documented [59].

In the present meta-analysis, we sought to estimate the association between malnutrition and risk of in-hospital death in adults hospitalized with COVID-19. Our study only included those studies published between 2021 and 2023, permitting an estimation of this association in patients hospitalized with legacy as well as current variants of the virus. It is clear that malnutrition is an ominous prognostic sign in patients hospitalized with COVID. This meta-analysis, which includes studies from nine countries on four continents with data from 350,021 patients, seems generalizable.

### 4.1. Limitations

A potentially important study limitation was the identification of publication bias in the pooled prevalence estimates of malnutrition. While none were included in the present meta-analysis, an examination of unpublished but completed studies in study registries identified four such projects, all inClinicalTrials.gov: Nutritional Assessment of Hospitalized Patients With COVID-19 (DenutCOVID), which recruited ninety participants and did not post results, and for which malnutrition was the outcome variable (NCT04503525); Minimizing the Effects of COVID-19 Hospitalization With the COVID Rehabilitation Program for the Elderly (CORE) enrolled 124 participants in a randomized clinical trial in which oral nutrition supplements were included to prevent malnutrition, but in-hospital mortality was not a study endpoint (NCT04771052); an observational study in which 986 participants were enrolled, Investigating the Role of Vitamin D in the Morbidity of COVID-19 Patients (NCT04386044) examined the role of blood vitamin D levels (but not overall nutrition status) on several clinical outcomes; and Increased Risk of Severe Coronavirus Disease 2019 in Patients With Vitamin D Deficiency (COVIT-D), another observational study with 300 enrolled patients also looked at vitamin D nutriture (but not overall nutrition status) on COVID-19 severity (NCT04403932). Another important limitation is the high degree of heterogeneity in the included studies. This was remedied by using the random effects model; however, it cannot be assumed that the heterogeneity was fully neutralized. Other limitations include between-study variability in patient mix; between-study variability in COVID-19 variant (variant was not reported in any of the studies); and between-study variability in nutrition status screening/malnutrition diagnosis. This overall heterogeneity was remedied by using the random effects model, but residual variability may still influence findings.

### 4.2. Implications

Findings of the present meta-analysis stress the importance in nutrition screening in adults hospitalized with COVID-19. While none of the included studies examined the impact of nutrition interventions, this is clearly an important future research objective. However, even without demonstrating efficacy, the successful use of nutrition interventions in a wide variety of clinical settings suggests that harm cannot be done to patients by improving their nutrition.

## 5. Conclusions

Malnutrition is a common finding in patients hospitalized with COVID-19 and greatly increases the odds of in-hospital death.

## Figures and Tables

**Table 1:** Summary of Included Studies.

Citation	Study Design	Number of Patients	Malnutrition Measure	Country	Publication Date
Zhang K, Gui H, Cong J, He P. A modified nutrition risk screening 2002 predicts the risk of death among hospitalized patients with COVID-19. Clin Nutr ESPEN. 2022; 52:365–370 [34].	Prospective	678	Modified NRS; NRS 2002; MNA-SF; MUST	China	Dec-22
Ponce J, Anzalone AJ, Bailey K, Sayles H, Timmerman M, Jackson M, McClay J, Hanson C; National COVID Cohort Collaborative (N3C) Consortium. Impact of malnutrition on clinical outcomes in patients diagnosed with COVID-19. JPEN J Parenter Enteral Nutr. 2022; 46: 1797–1807 [35].	Cross sectional	343,188	Diagnostic code in electronic medical record	USA	Nov-22
da Silva CL, Sousa TMM, de Sousa Junior JB, Nakano EY. Nutritional factors associated with mortality in hospitalized patients with COVID-19. Clin Nutr Open Sci. 2022; 45:17–26 [36].	Historical prospective	222	NRS 2002	Brazil	Aug-22
Gregoriano C, Voelkle M, Koch D, Hauser SI, Kutz A, Mueller B, Schuetz P. Association of Different Malnutrition Parameters and Clinical Outcomes among COVID-19 Patients: An Observational Study. Nutrients. 2022;14:3449 [37].	Prospective	305	NRS 2002; BMI; albumin	Switzerland	Aug-22
Shabanpur M, Pourmahmoudi A, Nicolau J, Veronese N, Roustaei N, Jahromi AJ, Hosseinikia M. The importance of nutritional status on clinical outcomes among both ICU and Non-ICU patients with COVID-19. Clin Nutr ESPEN. 2022; 49:225–231 [38].	Prospective	400	NRS 2002	Iran	Jun-22
Aktan A, Güzel T, Demir M, Özbek M. The effect of nutritional scores on mortality in COVID-19 patients. Rev Assoc Med Bras (1992). 2022; 68:1096–1102 [39].	Retrospective	1488	CONUT, PNI	Turkey	Aug-22
Nunes EC, Marcon S, Oliveira PE, Loss SH. Nutritional profile and outcomes of noncritical hospitalized patients with COVID-19 in a large tertiary hospital in southern Brazil. Rev Assoc Med Bras (1992). 2022; 68: 1216–1220 [40].	Cross sectional	526	NRS 2002, diet acceptance (% food consumed)	Brazil	Sep-22
Polat O, Yuruyen M, Sonmezoz GB, Kansu AD, Erismis B, Karendere F, Kocoglu H, Karabela S, Yasar KK. Malnutrition risk frequency and independent risk factors associated with mortality in hospitalized elderly patients with COVID-19 in Turkey. Asia Pac J Clin Nutr. 2022; 31:355–361 [41].	Cross sectional	451	NRS 2002	Turkey	Mar-22
Nicolau J, Ayala L, Sanchís P, Olivares J, Dotres K, Soler AG, Rodríguez I, Gómez LA, Masmiquel L. Influence of nutritional status on clinical outcomes among hospitalized patients with COVID-19. Clin Nutr ESPEN. 2021; 43:223–229 [42].	Cross sectional	75	SGA	Spain	Apr-21
Caccialanza R, Formisano E, Klersy C, Ferretti V, Ferrari A, Demontis S, Mascheroni A, Masi S, Crotti S, Lobascio F, Cerutti N, Orlandoni P, Dalla Costa C, Redaelli E, Fabbri A, Malesci A, Corrao S, Bordandini L, Cereda E; NUTRI-COVID19 Collaborative Working Group. Nutritional parameters associated with prognosis in non-critically ill hospitalized COVID-19 patients: The NUTRI-COVID19 study. Clin Nutr. 2022; 41:2980–2987 [43].	Prospective	1391	Self-reported reduction of food intake 3–5 days prior to hospitalization	Italy	Dec-22
Kananen L, Eriksdotter M, Boström AM, Kivipelto M, Annetorp M, Metzner C, Bäck Jerlardtz V, Engström M, Johnson P, Lundberg LG, Åkesson E, Sühl Öberg C, Hägg S, Religa D, Jylhävä J, Cederholm T. Body mass index and Mini Nutritional Assessment-Short Form as predictors of in-geriatric hospital mortality in older adults with COVID-19. Clin Nutr. 2022; 41:2973–2979 [44].	Retrospective	1297	MNA, BMI	Sweden	Dec-22
Vong T, Yanek LR, Wang L, Yu H, Fan C, Zhou E, Oh SJ, Szvarca D, Kim A, Potter JJ, Mullin GE. Malnutrition Increases Hospital Length of Stay and Mortality among Adult Inpatients with COVID-19. Nutrients. 2022;14:1310 [45].	Retrospective	4311	MUST	USA	Mar-22

**Table 2:** The impact of malnutrition on risk of in-hospital death.

Study	Sample Size	Proportion with Elevated Malnutrition Risk/Diagnosis of Malnutrition	Odds Ratio	95% CI Odds Ratio	% Weight Fixed	% Weight Random
Zhang et al. [34]	67/484	1/194	31.0	4.27–225.03	0.021	1.95
Ponce et al. [35]	7455/26,917	33,831/316,217	3.19	3.11–3.29	97.85	16.06
DaSilva et al. [36]	40/137	11/85	2.77	1.33–5.77	0.15	8.09
Gregoriano et al. [37]	20/76	24/229	3.05	1.57–5.92	0.19	8.89
Shabanpur et al. [38]	21/387	0/13	1.58	0.09–27.55	0.01	1.00
Aktan et al. [39]	316/1225	9/263	9.81	4.99–19.31	0.18	8.72
Nunes et al. [40]	26/107	5/419	26,58	9.91–71.2	0.084	5.76
Polat et al. [41]	65/292	13/159	3.22	1.711–6.04	0.21	9.29
Nicolau et al. [42]	2/27	0/48	9.51	0.44–205.69	0.009	0.87
Caccialanza et al. [43]	291/984	71/407	1.99	1.49–2.66	0.98	13.92
Kananen et al. [44]	126/2070	19/227	1.46	0.88–2.42	0.32	10.94
Vong et al. [45]	102/403	453/3908	2.59	2.02–3.30	1.35	14.48
Total (fixed effects)	8531/32,109	34,437/322,169	3.19	3.11–3.29	100.00	100.00
Total (random effects)	8531/32,109	34,437/322,169	3.43	2.59–5.46	100.00	100.00

Z < 0.001, *p* < 0.001 for both total fixed effects and total random effects models. Q = 56.49; *I*^2^ = 80.53%, 95% CI 66.91–88.54, *p* < 0.001. Egger’s test intercept = 0.21, 95% CI −1.47–1.89, *p* = 0.79. Begg’s test Kendall’s Tau = 0.24, *p* = 0.27.

**Table 3:** Elevated Malnutrition risk/Malnutrition Prevalence in Included Studies.

Study	Sample Size	Proportion with Elevated Malnutrition Risk/Diagnosis of Malnutrition	95% CI Odds Ratio	% Weight Fixed	% Weight Random
Zhang et al. [34]	678	71.39	62.82–74.76	0.19	8.35
Ponce et al. [35]	343,188	7.84	7.75–7.93	98.05	8.36
DaSilva et al. [36]	222	61.71	54.97–68.14	0.06	8.31
Gregoriano et al. [37]	305	24.92	20.16–30.17	0.09	8.33
Shabanpur et al. [38]	400	96.75	94.51–98.26	0.11	8.33
Aktan et al. [39]	1488	82.33	80.29–84.23	0.43	8.36
Nunes et al. [40]	526	20.34	16.98–24.04	0.15	8.34
Polat et al. [41]	451	64.75	60.14–69.16	0.13	8.34
Nicolau et al. [42]	75	36.00	25.23–47.91	0.02	8.21
Caccialanza et al. [43]	1391	70.74	68.27–73.12	0.40	8.36
Kananen et al. [44]	1297	82.49	80.32–84.53	0.37	8.36
Vong et al. [45]	4311	9.35	8.49–10.26	1.22	8.36
Total (fixed effects)	354,332	8.63	8.53–8.71	100.00	100.00
Total (random effects)	354,332	52.61	29.50–75.14	100.00	100.00

Q = 15,355.0902; *I*^2^ = 99.93, 95% CI 99.92–99.93. Egger’s intercept –31.82, 95% CI 15.09–48.55, *p* = 0.002. Begg’s test Kendall’s Tau = −0.12, *p* = 0.58.

## Data Availability

Data are presented herein.
